# *Pseudomonas aeruginosa* Biofilms in Cystic Fibrosis: Interactions, Methods, and Therapeutic Strategies

**DOI:** 10.1155/bmri/5328382

**Published:** 2026-02-26

**Authors:** Luis Ángel Núñez-García, Carlos Córdova-Fletes, María Carmen Barboza-Cerda, Elvira Garza-González

**Affiliations:** ^1^ Department of Biochemistry and Molecular Medicine, Faculty of Medicine, Autonomous University of Nuevo Leon, Monterrey, Nuevo Leon, Mexico, uanl.mx

## Abstract

This review explores the role of *Pseudomonas aeruginosa* biofilms in cystic fibrosis (CF) pathogenesis. Biofilms, the main bacterial lifestyle in CF lungs, are key in therapy failure, immune evasion, and chronic infection persistence. This review examines biofilm structure, emphasizing extracellular polymeric substances (Psl, Pel, alginate, eDNA) and their roles in structural stability, resistance to antibiotics, and immune modulation. Regulatory mechanisms, including c‐di‐GMP signaling and quorum‐sensing systems, are detailed as key drivers of biofilm formation and maintenance. The review also highlights polymicrobial interactions, particularly with *Staphylococcus aureus*, *Candida* spp., and *Aspergillus* spp., and commensal bacteria, illustrating how interaction dynamics shape microbial behavior, virulence, and treatment outcomes. Methods for studying biofilms in CF‐like conditions, such as advanced in vitro models and transcriptomic analyses, are outlined for their relevance in replicating the complex lung environment. Emerging antibiofilm strategies, including matrix‐disrupting enzymes, quorum‐sensing inhibitors, bacteriophage therapies, and nanomedicine, are discussed as promising tools to combat biofilm resilience. The review underscores the need for innovative therapeutic approaches and a deeper understanding of microbial and host interactions to improve clinical outcomes in CF patients.

## 1. Introduction

Cystic fibrosis (CF) is a hereditary disease that affects ~150,000 individuals globally [1]. Advances in medical care have significantly improved life expectancy for people with CF (pwCF) [2]. This extended lifespan has brought age‐related complications such as CF‐related diabetes, kidney disease, metabolic bone disease, and progressive lung function decline [3]. Among these, lung disease is the main cause of morbidity and mortality, particularly in older pwCF [4–6].

*Pseudomonas aeruginosa* is the most commonly isolated pathogen from adult pwCF [7] in which colonization often starts in early childhood, typically through environmental exposure to the bacteria [8]. Once established in the CF airway, *P. aeruginosa* becomes a lifelong resident, adapting genetically and phenotypically to the lung environment. These adaptations include the development of multidrug resistance, decreased growth rates, hypermutation, changed quorum sensing (QS), and a shift toward a biofilm lifestyle [9–13].

Biofilms are the dominant lifestyle of *P. aeruginosa* in CF lungs [12]. They provide enhanced resistance to antibiotics [14–16], protection from host immune responses [17], facilitate horizontal gene transfer, and enable coexistence among sublineages or with different species [18, 19].

Polymicrobial interactions within mixed‐species and mixed‐sublineage biofilms change antibiotic susceptibility [20, 21], bacterial metabolism, and production of virulence factors [22–24]. Biofilm structure and development vary based on the microbial species and even the strains within a species. CF lung biofilms are believed to form within the dense mucus layer, unlike the typical surface‐associated biofilm model [25].

Pathoadaptive changes shape the structure, localization, and resilience of bacterial aggregates in the lung [12]. The overproduction of alginate in mucoid phenotypes changes biofilm properties [19, 26, 27], and biofilm‐related exopolysaccharides contribute to treatment failures in CF [16, 28, 29]. Persistent inflammation in CF lungs recruits neutrophils that release extracellular traps (NETs) and contribute to biofilm rather than eliminating the bacteria [10].

This review provides a comprehensive overview of *P. aeruginosa* biofilms in the context of CF. It provides a framework for understanding how certain adaptations to the CF lung environment guide biofilm development and engage with other members of the lung microbiome. It also highlights biofilms as key therapeutic targets and discusses existing methods to study these structures under conditions that mimic the CF lung environment.

### 1.1. CF

CF is an autosomal recessive hereditary disease caused by mutations in the cystic fibrosis transmembrane conductance regulator (CFTR) gene, affecting approximately 150,000 individuals worldwide [1]. Defective CFTR function leads to impaired ion transport across epithelial cells, resulting in dehydration and accumulation of thick mucus in mucosecretory tissues [6]. In the lungs, this thickened mucus provides an optimal environment for microbial colonization, making pwCF susceptible to recurrent respiratory infections. Progressive lung damage driven by infections is the leading cause of mortality in this population [4]. Among the various microorganisms that colonize the CF airway, *P. aeruginosa* is the predominant pathogen in chronic infections [7] and is strongly associated with the progressive decline in pulmonary function [30, 31].

### 1.2. *P. aeruginosa*

*P. aeruginosa* is a Gram‐negative opportunistic pathogen characterized by encoding numerous virulence factors, intrinsic resistance to a broad spectrum of antibiotics [32, 33], and metabolic versatility, allowing survival across diverse environments [34]. The adaptability of *P. aeruginosa* favors colonization and infection in a wide range of hosts, including mammals and plants [35]. In CF, *P. aeruginosa* undergoes pathoadaptive changes such as enhanced biofilm formation, diminished motility, and a shift to mucoid phenotypes characterized by high levels of alginate production [36]. These adaptations promote immune evasion and enable persistence despite chronic inflammatory responses [10].

Strains with adaptive mutations frequently exhibit reduced motility, O‐antigen biosynthesis [37], growth rate [38], and elastase production [39]. The loss of surface structures like pili, flagella, and O‐antigen contributes to immune evasion [37]. Additionally, slower growth rates [40] and biofilm formation confer increased resistance to antibiotics [19, 41, 42]. The mucoid switch, characterized by excessive alginate production, further interferes with the penetration of antibiotics into biofilms [10, 11].

### 1.3. Components of the Biofilm Extracellular Matrix (ECM) in *P. aeruginosa*

Biofilms are structured microbial communities embedded in an ECM, often associated with a surface. Biofilms of *P. aeruginosa* CF strains are of particular interest due to their role in therapy failure, immune evasion, and persistence in chronic infections [17] (Figure 1).

Figure 1Role of *P. aeruginosa* biofilm matrix components. (a) Nonmucoid biofilms: Primarily composed of the exopolysaccharides Psl, Pel, and eDNA. Within the biofilm, eDNA can be used as a source of carbon, nitrogen, and phosphate through endogenous DNase activity. Pel and eDNA form a complex driven by opposing charges, which both protect eDNA from DNase degradation and reduce the permeability of tobramycin. Psl mediates tighter matrix packing, further limiting antibiotic penetration and promoting interspecies interactions. (b) Mucoid biofilms: In mucoid strains, alginate becomes the dominant matrix component. Cross‐linking between alginate and calcium ions increases the biofilm’s surface‐to‐volume ratio and decreases both phagocytosis and permeability to antibiotics and the LL‐37 antimicrobial peptide. Alginate also interferes with the PQS quorum sensing pathway. Mucoid revertants within the biofilm secrete catalase, which confers increased resistance to oxidative stress by reducing susceptibility to hydrogen peroxide.

**Figure:**
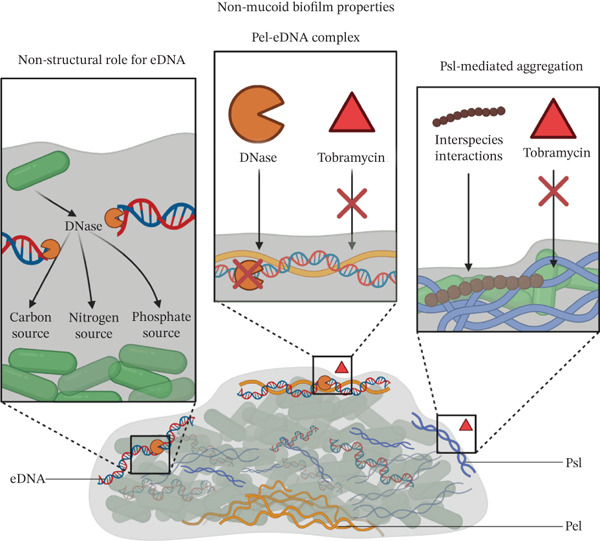
(a)

**Figure:**
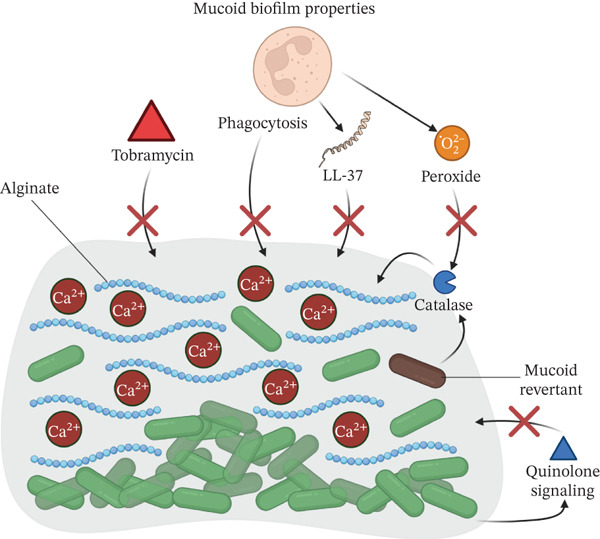
(b)

The ECM of biofilms is a complex assembly of substances produced by *P. aeruginosa* and components incorporated from the surrounding environment and, in some cases, interactions with other microbes. The ECM constitutes approximately 90% of the biofilm biomass, with extracellular polymeric substances (EPSs) as its primary constituents. EPSs are the primary drivers of antibiotic resistance in biofilms compared to planktonic cells. The four major EPS components of the *P. aeruginosa* biofilms are Psl, Pel, alginate, and extracellular DNA (eDNA) [43].

#### 1.3.1. Psl

Psl is a neutral pentasaccharide composed of L‐rhamnose, D‐glucose, and three units of D‐mannose; Psl has a high‐molecular‐weight (cell‐associated) and low‐molecular‐weight (soluble) form [44, 45]. It plays a key role in the initial stages of biofilm development, mediating bacterial attachment in a helical arrangement [46]. During biofilm maturation, Psl localizes to the periphery, where it cross‐links with eDNA and the CdrA protein to provide structural stability [43, 44, 46, 47]. Beyond structure, Psl acts as a signaling molecule that coordinates microcolony development and activates the synthesis of cyclic‐di‐GMP (c‐di‐GMP) [43]. Psl also triggers the host immune system to produce reactive oxygen species (ROS) and protects bacteria from phagocytosis, opsonization, and antibiotics [10, 28, 29, 43].

Stoner et al. described a role for Psl in polymicrobial biofilm formation, where Psl‐mediated interactions with commensal species like *Streptococcus salivarius* strengthened the dual‐species biofilm development through maltose‐binding protein MalE. Such interactions may influence the dynamics and pathogenicity of microbial communities in CF airways [48]. Psl is implicated in eradication therapy failures; Morris et al. showed that strains resilient to therapy show higher anti‐Psl binding in vitro and were more resistant to tobramycin compared with strains that were successfully eradicated [28]. The same research group further demonstrated higher Psl‐binding aggregates in sputum samples from the same patient cohort, suggesting a role in aggregation and antibiotic resistance [29].

#### 1.3.2. Pel

Pel is a cationic polymer composed of partially acetylated N‐acetyl‐galactosamine and N‐acetyl‐glucosamine in a 5:1 ratio; Pel also exists in cell‐associated and secreted forms [46]. It primarily contributes to the structural stiffness and roughness of biofilms through pellicle formation, increasing surface‐to‐volume ratios [45, 46]. Pel has major roles during the early stages of biofilm development and is essentially absent during biofilm maturation; it thus tends to localize to the stalk [46, 47]. Pel can replace Psl functions in mutants lacking the latter and hence localize to the periphery in those circumstances. The high binding affinity for eDNA, mediated by its opposing charges, greatly contributes to biofilm resistance against DNAse digestion and also cationic antibiotics, like tobramycin [44, 45]. Furthermore, in support of these observations, research has identified Psl‐ and Pel‐positive aggregates directly in sputum, where the interaction between Pel and eDNA confers resistance to tobramycin and protects eDNA against DNAse degradation [16].

#### 1.3.3. Alginate

Alginate consists of partially O‐acetylated units of *β*‐D‐mannuronate and *α*‐L‐guluronate and is overproduced by mucoid *P. aeruginosa* strains [47]. It forms a viscoelastic hydrogel, which retains water and contributes to biofilm stickiness [44, 45]. Alginate is negatively charged, which promotes binding of positive ions, such as calcium, which cross‐link the alginate matrix and enhance antibiotic resistance [46]. CF strains often undergo a mucoid switch characterized by overproduction of the polysaccharide alginate that confers a selective advantage in the dense mucus environment of CF lungs.

The overproduction of alginate is attributed to loss‐of‐function mutations in the transcriptional regulator *mucA*; these mutations prevent interaction with the AlgT/U sigma factor, leading to upregulated alginate synthesis [10]. In mucoid phenotypes, alginate becomes the dominant exopolysaccharide, significantly influencing biofilm properties.

A study examining the in vitro properties of mucoid biofilms in the presence of calcium showed that mucoid strains are capable of biofilm development independently of Psl, Pel, CdrA, and c‐di‐GMP stimulation. Additionally, calcium–alginate interactions, combined with eDNA binding, increased biofilm viscoelasticity [27] and interfered with neutrophil phagocytosis [49].

Alginate also protects *P. aeruginosa* and the surrounding biofilm communities from the antimicrobial peptide (AMP) LL‐37 produced by neutrophils [19]. High concentrations of alginate, however, interfere with QS signaling, particularly the Pqs system discussed below in this review [26]. In some cases, the mucoid phenotype can revert through secondary mutations in *algT/U*, which also leads to increased catalase synthesis and resistance to H_2_O_2_‐mediated oxidative stress; this effect brings a protective role to neighboring cells within the biofilm [19].

#### 1.3.4. eDNA

eDNA is a versatile component of the ECM. It is liberated through autolysis of *P. aeruginosa,* added by dead cells, or by NETs secreted by the host immune system [10]. eDNA serves as a carbon, nitrogen, and phosphate nutrient source [50], as a structural element, and as a mechanism for horizontal gene transfer and DNA repair [46]. It can also interact with Psl and Pel to form DNAse‐resistant complexes and impede the penetration of antibiotics, enhancing the resilience of biofilms [43, 46].

### 1.4. Biofilm Development and Regulation

Biofilm formation progresses through five key stages: reversible attachment, irreversible attachment, two distinct maturation steps (clustering and microcolony formation), and dispersion [51]. Its development is regulated by two main mechanisms: c‐di‐GMP signaling and QS systems. Levels of c‐di‐GMP regulate the transition from a motile state, characterized by basal levels of c‐di‐GMP, to a biofilm matrix production and adhesion state, with high intracellular levels of c‐di‐GMP [52]. On the contrary, QS molecules enable groups of bacteria to coordinate activation of specific pathways as a response to population density sensing and thus depend on the amount of bacteria on a given medium [53].

#### 1.4.1. c‐di‐GMP

c‐di‐GMP is a second messenger that shifts the behavior of *P. aeruginosa* from a motile planktonic state to a biofilm‐producing state. Its levels are regulated by diguanylate cyclases (DGCs), which synthesize c‐di‐GMP from GTP, and phosphodiesterases (PDEs), which degrade it to c‐GMP [44, 54]. Environmental stimuli, such as light, nitric oxide, oxygen, and mechanical forces, induce the activation of DGCs, leading to EPS production and biofilm maturation [52]. Elevated levels of c‐di‐GMP then promote exopolysaccharide synthesis, an essential step in the initial stages of attachment due to the rapid activation of the DGC activity of WspR via the surface‐sensing property of WspA. Biofilm development furthermore continues by maintaining elevated levels of c‐di‐GMP via multiple DGCs at each stage; these are considered to conduct nonoverlapping functions [54]. After the biofilm maturation phase, factors such as nitric oxide, glutamate, and oxygen limitation induce the dispersal of biofilm, where PDEs take a leading role over DGCs [52].

#### 1.4.2. QS

QS is a cell density–dependent communication mechanism that regulates bacterial behavior, including virulence and biofilm formation. QS systems rely on the production and detection of small signaling molecules, known as autoinducers, which are recognized by cognate receptor proteins that function as transcriptional regulators. *P. aeruginosa* has four recognized QS systems: Las, Rhl, Pqs, and IQS, which are interconnected hierarchically [55].

The *Las system* relies on the signal molecule N‐3‐oxo‐dodecanoyl homoserine lactone (3‐oxo‐C12‐HSL) and the LasR transcriptional regulator. It is the top‐level QS system and regulates genes involved in biofilm formation, such as those encoding extracellular enzymes and polysaccharides [56]. The *Rhl system* uses N‐butanoyl‐homoserine lactone (C4‐HSL) and RhlR. It operates downstream of the Las system and is associated with rhamnolipid production, which facilitates biofilm dispersion and the maintenance of biofilm architecture [57]. The *Pqs system* involves 2‐heptyl‐3‐hydroxy‐4‐quinolone (PQS) and PqsR (MvfR). It is unique to *P. aeruginosa* and regulates genes involved in eDNA release and iron acquisition, both critical for biofilm formation [58]. Finally, the *IQS system* involves the production of the pyochelin‐derived molecule 2‐(2‐hydroxylphenyl)‐thiazole‐4‐carbaldehyde, whose cognate receptor is still unknown. This system is thought to be involved in biofilm formation inhibition/dispersal [59].

QS plays a dual role in biofilms: It promotes biofilm maturation through EPS production and eDNA release and facilitates dispersal by triggering rhamnolipid synthesis. In CF, QS signaling is often dysregulated due to mutations in QS‐related genes, leading to a loss of virulence factor production but enhanced biofilm persistence [60].

Other molecules, such as the Rhl‐controlled phenazine production, affect biofilm morphology. The reduced phenazine produced by the *psl*‐deficient *P. aeruginosa* strain PA14 leads to a structured and rough biofilm, while overproduction of phenazines developed into a smoother‐structured biofilm [55]. Other QS‐regulated molecules affecting biofilm architecture are rhamnolipids, controlled through the Rhl system. Their amphiphilic properties facilitate interactions with exopolysaccharides, promoting water channel formation and contributing to biofilm dispersal in later stages [45, 56]. Additionally, the LasA regulon positively influences biofilm development by regulating the *pel* operon, further underscoring the complex interplay between QS systems and biofilm architecture [57].

The Pqs system also impacts biofilm development. Disruption of *pqsR* reduces biofilm biomass, colony‐forming unit (CFU) recovery, and biofilm‐associated antibiotic resistance [58]. This system influences eDNA release through 2‐heptyl‐4‐hydroxyquinoline N‐oxide (HQNO)–dependent autolysis, a process fundamental to biofilm ECM integrity [60]. Similar effects are observed in *lasR* mutants, likely due to LasR‐mediated Pqs activation [61], underscoring the interconnectedness of QS systems. Interestingly, the *lasR* mutations that are common in *P. aeruginosa* isolates from pwCF shift the QS regulation to the Rhl system as the major driver of QS‐regulated gene expression, underlying the adaptive plasticity of QS systems in response to environmental pressures and host conditions [62].

### 1.5. Persistence of *P. aeruginosa* in pwCF

Establishment of *P. aeruginosa* in the CF airway is associated with adaptive genetic and phenotypic adaptations to this environment. Clonal dominance, decreased antibiotic susceptibility, reduced growth rates, hypermutation, altered QS, and a transition to a biofilm lifestyle with high alginate production are some of the common adaptations [9–13]. Furthermore, phenotypic heterogeneity is a major factor involved in persistent infections of *P. aeruginosa*. This heterogeneity arises from gradients of nutrients, oxygen, waste products, and signaling molecules across the biofilm structure, leading to the appearance of persister cells and viable but nonculturable (VBNC) subpopulations. These distinct physiological states can survive antibiotic treatment and subsequently reestablish infection [63, 64].

Clonal dominance is commonly reported in CF airways [11]. Though strain replacement has been noted [65, 66], one strain often persists despite the introduction of unrelated strains unable to outcompete the established strain [67]. Interstrain competition mechanisms are complex and understudied. Phage‐like bacteriocins (named pyocins in *P. aeruginosa*) play a determining role in clonal dominance within biofilms. A 2009 study showed that F‐ and R‐type pyocins are overproduced under anoxic conditions in in vitro biofilms. The release of pyocins led to the eradication of pyocin‐susceptible strains, either in cocultures or in cell‐free supernatants [68]. Recent findings suggest that pyocin production alone dictates strain dominance in mixed‐strain biofilms derived from CF [69]. Sublethal concentrations of pyocins induce an increase in biofilm formation in susceptible strains through mechanisms similar to those induced by antibiotics, which cause cell wall stress [70]. Furthermore, strains isolated from pwCF are mostly susceptible to R2‐type pyocins [71], which corresponds with the finding that R1‐type producers tend to dominate these environments [72].

## 2. Polymicrobial Interactions of *P. aeruginosa*

Polymicrobial interactions in the CF lungs influence infection dynamics, persistence, and therapeutic outcomes. These interactions range from competition to cooperation and drive the behavior of *P. aeruginosa* and its biofilm formation capabilities. Understanding such relationships is of paramount importance in developing therapeutic strategies, as the CF lung microbiome is a dynamic environment with multiple bacterial and fungal species.

Besides *P. aeruginosa*, pathogens of CF include *Staphylococcus aureus*, *Haemophilus influenzae*, *Burkholderia* spp., *Stenotrophomonas maltophilia*, and *Achromobacter* spp. The CF lung microbiome includes species of the *Streptococcus*, *Prevotella*, *Rothia*, *Veillonella*, and *Haemophilus* genera [30, 31, 73–75]. Common fungal species are *Candida* spp., *Aspergillus* spp., *Exophiala* spp., and *Scedosporium* spp. [76, 77]. Microbial diversity has gained increasing relevance in studies of *P. aeruginosa* behavior (Table 1). However, most studies fail to accurately represent this diversity in in vitro biofilm formation [20].

**Table 1:** Summary of reports studying the interaction between *P. aeruginosa* and common residents of the CF lung (N/A: not applicable; N/T: not tested; CFBE: cystic fibrosis bronchial epithelial cells; TSB: trypticase‐soy broth media; LB: Luria–Bertani; YPD: yeast extract–peptone–dextrose media; SCFM2: synthetic cystic fibrosis media 2; PYO: pyocyanin; BHI: blood–heart infusion media; MEM: minimum essential media).

Molecule	Strains	Culture conditions	Culture time (h)	Effect on interaction	Effect on susceptibility	Underlying mechanism	Reference
HQNO	*S. aureus* LAC and *P. aeruginosa* PA14	SCFM2 biofilms with agitation	16	Inhibition of *S. aureus*	N/T	Interference with respiratory metabolism	[78]
		SCFM2 static biofilms	16	Loss of HQNO‐mediated inhibition of *S. aureus*	Increased resistance to tobramycin of *S. aureus* in coculture with HQNO‐producing *P. aeruginosa* compared to non‐HQNO producing bacteria		
HCN	Reference and clinical *S. aureus* and *P. aeruginosa* isolates	Biofilm microfermentor with LB broth	48	Inhibition of *S. aureus*	N/T	Interference with respiratory metabolism	[79]
		SCFM2 static biofilms	22				
		BALB/c mouse lung infection	24				
HQNO and pyoverdine	*S. aureus* USA300 and *P. aeruginosa* PA14	*S. aureus* biofilm monoculture on MEM exposed to *P. aeruginosa* supernatants	26	No effect	Increased resistance of *S. aureus* to vancomycin	Interference with respiratory metabolism	[80]
		CFBE *S. aureus* monoculture exposed to *P. aeruginosa* supernatants	21	No effect	Increased resistance of *S. aureus* to vancomycin		
		Anoxic *S. aureus* monoculture	26	N/A	Increased resistance of *S. aureus* to vancomycin		
		CFBE coculture	24	Inhibition of *S. aureus*	Decreased survival		
HQNO, pyoverdine, HCN, LasA, and rhamnolipids	*S. aureus* HG003 and multiple reference and clinical *P. aeruginosa* isolates	Aerobic and anaerobic planktonic *S. aureus* monocultures exposed to *P. aeruginosa* aerobic supernatants	24	N/T	Increased resistance of *S. aureus* to ciprofloxacin and tobramycin	Inhibition of respiratory metabolism by HQNO, HCN, and PYO	[81]
		Murine burn injury	48	N/T	Increased killing of *S. aureus* by tobramycin	Rhamnolipids induce higher antibiotic uptake	
					Increased killing of *S. aureus* by vancomycin	LasA‐mediated lysis	
LasA, LasB, pyocyanin, pyoverdine, and rhamnolipids	*S. aureus* ATCC 6538 and *P. aeruginosa* PAO1 + clinical isolate	Aerobic planktonic and biofilm cocultures in LB broth	16	Inhibition of *S. aureus*	N/T	Anaerobiosis decreases the production of virulence factors of *P. aeruginosa*, an unidentified molecule showed an inhibitory effect	[82]
		Anaerobic planktonic and biofilm cocultures in LB broth	16	Inhibition effect over *S. aureus* lost	N/T		
Alginate	*S. aureus* JE2 and Newman; multiple reference and clinical *P. aeruginosa* isolates	Planktonic and biofilm TSB, SCFM, and CFBE	24	Coexistence between *S. aureus* and *P. aeruginosa* is promoted by alginate overproduction	N/T	Decreased production of HQNO, siderophores, and rhamnolipids in *P. aeruginosa* overproduction of alginate	[83]
Acetoin	Reference and clinical *S. aureus* and *P. aeruginosa* isolates	Planktonic cocultures with agitation in BHI broth	8	Coexistence of *S. aureus* and *P. aeruginosa*	N/T	Cross‐feeding of acetoin secreted by *S. aureus* and used as a carbon source by *P. aeruginosa*	[84]
Acetoin and acetic acid	Reference and clinical *S. aureus* and *P. aeruginosa* isolates	Planktonic cocultures of *P. aeruginosa* and *S. aureus* with agitation in TSB and *P. aeruginosa* grown in TSB + supernatants from *S. aureus*	24	*S. aureus* inhibits *P. aeruginosa*	N/T	Byproducts of glucose metabolism secreted by *S. aureus* inhibit *P. aeruginosa* by a pH‐dependent mechanism	[85]
Alkyl‐quinolones and siderophores	Reference and clinical *S. aureus* and *P. aeruginosa* isolates	*S. aureus* planktonic culture exposed to *P. aeruginosa* TSB supernatants and static Transwell coculture in TSB	18	*P. aeruginosa* inhibits *S. aureus*	N/T	Iron depletion enhances respiratory inhibition‐dependent antimicrobial effects of alkyl quinolones	[86]
Cis‐2‐decenoic acid	*S. aureus* ATCC 12602	*S. aureus* microchamber‐grown biofilms in EPRI medium exposed to *P. aeruginosa* chloroform supernatant extracts	Dispersion induction for 1 h	*P. aeruginosa* induces *S. aureus* biofilm dispersal	N/T	N/T	[87]
Type IV pili	*S. aureus* USA300 JE2 and *P. aeruginosa* PA14	Static coculture biofilms in ASM	24	*P. aeruginosa* invades *S. aureus* biofilms and enhances antimicrobial effects	N/T	Type IV pili–directed chemotaxis enhances biofilm invasion and secretion of antimicrobial compounds of *P. aeruginosa* against *S. aureus*	[88]
Alginate	*S. aureus* JE2 and multiple *P. aeruginosa* clinical and reference isolates	Planktonic coculture with agitation in MEM	24	*P. aeruginosa* downregulates anti‐staphylococcal molecules production	N/T	Alginate downregulates the production of HQNO, siderophores, and rhamnolipids of *P. aeruginosa*	[42]
Spa/Psl	Clinical *S. aureus* and *P. aeruginosa* isolates	Static biofilm cultures of *P. aeruginosa* in LB broth treated with *S. aureus* culture supernatants	48	Increased aggregation of *P. aeruginosa* biofilms	Increased resistance of *P. aeruginosa* to tobramycin	*S. aureus* protein A (Spa) interacts with Psl from *P. aeruginosa* to promote biofilm packing	[89]
Lactate	*S. aureus* ATCC25923 and *P. aeruginosa* UCBPP‐PA14	Coculture biofilms of *P. aeruginosa* and *S. aureus* in TSB	24	Probable lactate and action cross‐feeding between *S. aureus* and *P. aeruginosa*	N/T	Upregulation of lactate and acetoin metabolism in *P. aeruginosa* and lactate metabolism in *S. aureus*	[90]
*Candida albicans* cell wall polysaccharides	Reference strains and clinical isolates of *Candida* spp. and *P. aeruginosa*	Static coculture biofilms of *Candida* spp. and *P. aeruginosa* in BHI	42	N/T	Increased tolerance of *P. aeruginosa* to meropenem	Mannans and glucans from *Candida* spp. protect *P. aeruginosa* from meropenem by an unknown mechanism	[91]
Unknown	Clinical isolates of *Candida* spp. and *P. aeruginosa*	Static *P. aeruginosa* monocultures exposed to *Candida* spp. supernatants	48	N/T	Increased tolerance of *P. aeruginosa* to meropenem and increased susceptibility for ceftazidime, ciprofloxacin, colistin, and tobramycin	Unknown	[92]
Farnesol	*P. aeruginosa* PA14 and *C. albicans* clinical isolate	Coculture of *P. aeruginosa* and *C. albicans* in YPD agar plates	24	Production of virulence factors of *P. aeruginosa*	N/T	Farnesol produced by *C. albicans* stimulates the production of PQS, phenazines, and C4‐HSL in LasR‐deficient *P. aeruginosa*	[22]
LasB elastase	*P. aeruginosa* PAO1 and PA14 + clinical isolates, and *A. fumigatus* clinical isolate	*P. aeruginosa* grown in methanol‐killed *A. fumigatus* biofilms in LB broth and epithelial cells	24	*P. aeruginosa* inhibits *A. fumigatus* and enhances cytotoxicity	N/T	Upregulation of *P. aeruginosa* LasB in contact with *A. fumigatus*	[93]
Phenazines and pyoverdine for *P. aeruginosa*; gliotoxin and siderophores for *A. fumigatus*	*P. aeruginosa* PA14 and *A. Fumigatus* clinical isolate	Aerobic and anaerobic static biofilm coculture of *P. aeruginosa* and *A. fumigatus* in RPMI	120	*P. aeruginosa* inhibits *A. fumigatus*, which is enhanced in hypoxia	N/T	Inhibition of *A. fumigatus* by *P. aeruginosa* phenazines leads to gliotoxin and siderophore production	[94]
Phenazines	Clinical isolates of *A. fumigatus* and *P. aeruginosa*	Biofilm monocultures of *A. fumigatus* in 2YT media	20	*P. aeruginosa* phenazines inhibit *A. fumigatus*	N/T	Phenazines produced by *P. aeruginosa* interfere with mitochondrial respiration and induce ROS production	[95]
Pyoverdine	Clinical and reference isolates of *P. aeruginosa* and *A. fumigatus* 10AF	Live cells, planktonic filtrate, and biofilm filtrate of *P. aeruginosa* vs. *A. fumigatus* in RPMI agar	24	*P. aeruginosa* pyoverdine inhibits *A. fumigatus*	N/T	Pyoverdine secreted by *P. aeruginosa* induces iron starvation in *A. fumigatus*	[96]
Pyoverdine, elastase, and rhamnolipids	Reference isolates of *P. aeruginosa* and *A. fumigatus*	Culture filtrates and coculture of *P. aeruginosa* vs. *A. fumigatus* in RPMI agar	48	*P. aeruginosa* elastase and rhamnolipids inhibit *A. fumigatus*	N/T	Iron depletion enhances the quorum sensing–dependent production of elastase and rhamnolipids of *P. aeruginosa*	[97]
Dimethyl sulfide	*P. aeruginosa* PAO1 and *A. fumigatus* CBS144‐9	Plate‐in‐plate cultures of *P. aeruginosa* and *A. fumigatus*	48	Sulfur‐containing molecules stimulate *A. fumigatus* growth	N/T	*A. fumigatus* uses volatile organic sulfur compounds secreted by *P. aeruginosa* to promote growth under sulfur starvation conditions	[98]
Secreted factors of *R. mucilaginosa*	Reference and clinical isolates of *P. aeruginosa* and *R. mucilaginosa*	Coinfection of cell cultures and in vivo mouse infection models	24	*R. mucilaginosa* decreases *P. aeruginosa-*induced inflammation	N/T	Secreted factors of *R. mucilaginosa* inhibit activation of the Nf‐*κ*B pathway and proinflammatory cytokines	[99]
HemE and an unidentified RNA‐binding protein in *S. mitis*	*P. aeruginosa* PAO1 and clinical isolates of *Streptococcus* spp.	Coinfection of cell cultures and precision cut lung slices from mice	8	Specific strains of *S. mitis* decrease *P. aeruginosa*‐induced inflammation	N/T	*S. mitis* downregulates the mTOR signaling pathway and NET formation	[100]
Secreted factors of *Prevotella* spp.	Clinical isolates of *P. aeruginosa* and *Prevotella* spp.	Coinfection of cell cultures	6	*Prevotella* spp.–secreted factors inhibit *P. aeruginosa* and reduce inflammatory markers in epithelial cells	N/T	Secreted factors of *Prevotella* spp. dampen TLR4 and Nf‐*κ*B signaling in epithelial cells and inhibit *P. aeruginosa*	[101]
Secreted factors of *Prevotella* spp.	Reference and clinical isolates of *P. aeruginosa* and clinical isolates *Prevotella* spp.	*P. aeruginosa* biofilms in BHI with agitation exposed to *Prevotella* spp. supernatants	24	*Prevotella* spp. isolates inhibit *P. aeruginosa* biofilm formation, growth rate, and protease secretion	N/T	Secreted factors of *Prevotella* spp. inhibit *P. aeruginosa* aggregation and biofilm matrix component production	[102]
Extended spectrum *β*‐lactamases	*P. aeruginosa* ATCC 27853 and clinical isolates of *Prevotella* spp.	Static coculture of *P. aeruginosa* and *Prevotella* spp. in Mueller–Hinton broth	48	N/T	Increased survival of *P. aeruginosa* against antibiotics when cocultured with *Prevotella* spp.	Production of *β*‐lactamases by *Prevotella* spp. shields *P. aeruginosa*	[103]
Acetate	*P. aeruginosa* PAO1 and clinical isolates of *Streptococcus* spp.	Shaken cocultures of *P. aeruginosa* and *Streptococcus* spp. in Columbia Broth	30	Specific strains of *Streptococcus* spp. inhibit *P. aeruginosa*	N/T	Acetate secreted by *Streptococcus* spp. disrupts the metabolism of *P. aeruginosa*	[104]
Lactate	Clinical and reference isolates of *P. aeruginosa* and *R. mucilaginosa*	*P. aeruginosa* culture in ASM exposed to *R. mucilaginosa* aerobic and anaerobic supernatants	24	Cross‐feeding between *P. aeruginosa* and *R. mucilaginosa*	N/T	Lactate secreted by *R. mucilaginosa* is used by *P. aeruginosa* as carbon source	[105]

A decrease in microbiome diversity is linked to poor clinical outcomes and reduced lung function, especially when *P. aeruginosa* dominates the microbial community [30, 31]. Chronic establishment of *P. aeruginosa* correlates with reduced microbial diversity and progressive pulmonary function decline [106]. Understanding *P. aeruginosa* interactions within the CF lung microbiome is essential for elucidating its biofilm behavior and responses to therapeutic interventions.

### 2.1. Interactions With *S. aureus*

**Figure 2:**
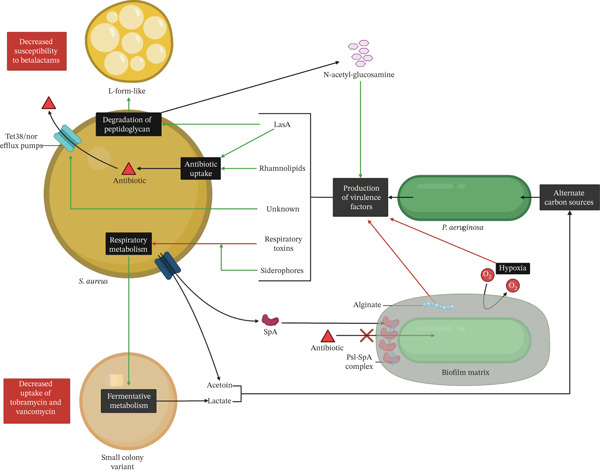
Overview of interactions between *P. aeruginosa* and *S. aureus*. Respiratory toxins from *P. aeruginosa* drive *S. aureus* toward small‐colony variant phenotypes characterized by fermentative metabolism and reduced antibiotic uptake, an effect enhanced by *P. aeruginosa* siderophores. Rhamnolipids secreted by *P. aeruginosa* increase *S. aureus* membrane permeability, leading to higher antibiotic uptake. The LasA (staphylolysin) protein cleaves *S. aureus* peptidoglycan, releasing N‐acetyl‐galactosamine that upregulates *P. aeruginosa* virulence factors and makes *S. aureus* more susceptible to vancomycin; this interaction can trigger L‐form‐like states in *S. aureus*, which show decreased susceptibility to *β*‐lactams. Additionally, *P. aeruginosa*–*S. aureus* cocultures can upregulate *S. aureus* efflux systems, while *P. aeruginosa* alginate production dampens its virulence factor expression. Staphylococcal protein A (SpA) complexes with the *P. aeruginosa* exopolysaccharide Psl, promoting tighter aggregation and further decreasing antibiotic uptake. Red arrows indicate a process being downregulated, and green arrows indicate a process being promoted. HQNO: 2‐heptyl‐4‐hydroxyquinoline N‐oxide; HCN: hydrogen cyanide; PYO: pyocyanin; PVD: pyoverdine; PCH: pyochelin.

One of the most extensively studied polymicrobial interactions in the CF lung is that of *P. aeruginosa* and *S. aureus*, which are frequently coisolated from the CF airways and other infection sites [23]. Environmental factors like oxygen availability, nutrient levels, and biofilm composition shape these interactions and have implications for bacterial adaptation and disease progression [107, 108]. This interaction is complex and multifaceted, with both competitive and coexistence behaviors (Figure 2).

#### 2.1.1. Competitive Interactions

*P. aeruginosa* competes with *S. aureus* by producing various antimicrobial compounds like the secondary metabolites pyocyanin (PYO), hydrogen cyanide (HCN), and HQNO. These compounds inhibit respiration in *S. aureus* [78–81, 86], favoring a transition to small‐colony variant (SCV) phenotypes characterized by a fermentative metabolism [24, 109]. *P. aeruginosa* also produces staphylolysin (LasA), which kills *S. aureus* by degrading the pentaglycine bridges in peptidoglycan. This can induce an L‐form‐like state in *S. aureus*, with reduced peptidoglycan synthesis and evasion of immune detection [109]. Furthermore, N‐acetyl‐glucosamine released by LasA‐mediated degradation of peptidoglycan enhances virulence factor synthesis in *P. aeruginosa* [24]. Other compounds with a hydrophobic nature, such as rhamnolipids and cis‐2‐decanoic acid, disperse biofilms of various species, including *S. aureus* [87, 110], and increase membrane permeability to antibiotics [81].

Twitching motility, mediated through type IV pili, facilitates antagonistic interactions, as *P. aeruginosa* can invade and reorganize the biofilms of *S. aureus*, promoting competition in a contact‐dependent way [88]. These interactions are also influenced by iron availability. *P. aeruginosa* produces the siderophores, pyoverdine and pyochelin, which enhance the effects of HQNO and related respiration‐inhibitory molecules [80, 86, 110]. Moreover, volatile compounds, such as HCN produced by *P. aeruginosa*, can inhibit the growth of *S. aureus* in a contact‐independent way [79].

#### 2.1.2. Transition to Coexistence Interaction

The dynamics of interaction between *P. aeruginosa* and *S. aureus* change in the hypoxic environment of the CF lung. For example, under anoxic conditions, mimicking CF sputum, the production of *P. aeruginosa* virulence factors like PYO and rhamnolipids is reduced, enhancing the coexistence with *S. aureus* [82]. Similarly, Barraza and Whiteley reported that HQNO‐dependent antagonism against *S. aureus* by *P. aeruginosa* is diminished under static biofilm conditions due to limiting oxygen availability. Moreover, the mutations that induce the transition of *P. aeruginosa* to a mucoid phenotype frequently disrupt HQNO, pyoverdine, and rhamnolipids biosynthesis, favoring coexistence [83]. Also, the expression of virulence factors is downregulated by alginate itself, independent of mutational events that affect its synthesis [42]. Collectively, these features of the CF lung environment enable metabolic and phenotypic transitions that favor decreased levels of interference directed against *S. aureus* during chronic infection, which might contribute to polymicrobial infections in vivo.

Interestingly, competitive interactions between the two species often collaterally benefit *S. aureus* by decreasing antibiotic susceptibility. The metabolic shift toward a fermentative metabolism mediated by respiratory toxins of *P. aeruginosa* inhibition promotes resistance of *S. aureus* to antibiotics like tobramycin [81] and vancomycin [80]. Moreover, exposure to *P. aeruginosa* upregulates the resistance‐associated *tet38* and *nor* gene families in *S. aureus* and triggers internalization in the epithelial cells at higher rates [111, 112]. Interaction between *S. aureus* protein A and *P. aeruginosa* Psl polysaccharide in mixed‐species biofilms increases biofilm density [89], limiting penetration of antibiotics [29]. Also, mature *S. aureus* biofilms can aid in the initial attachment of *P. aeruginosa* during the initial stages of cocolonization [112]. Consistent with the thought of competitive interactions enhancing in vivo persistence, one study showed that *P. aeruginosa* and *S. aureus* strains with a coexistence interaction phenotype had shorter cocolonization periods compared with strains having competitive interactions [111].

Trophic interactions also influence *S. aureus* and *P. aeruginosa* coexistence. Lactate generated by the fermentative metabolism of SCVs of *S. aureus* serves as an alternative carbon source for *P. aeruginosa* under nutrient‐limiting conditions [24]. In this context, *P. aeruginosa* upregulates genes involved in lactate transport and metabolism in in vitro biofilm cocultures [90].

Acetoin (3‐hydroxybutanone) secreted by *S. aureus* is also involved in trophic interactions, although the influence of this compound in competitive or coexistence states is less well defined. Camus et al. reported that acetoin can promote coexistence by serving as an alternative carbon source for *P. aeruginosa*, while simultaneously mitigating its toxic effects against *S. aureus* [84]. On the other hand, Kvich et al. reported a pH‐dependent inhibition of *P. aeruginosa* by acetoin and acetic acid [85]. These discrepancies may be attributed to differences in incubation times, as competitive interactions are more likely to emerge during extended incubation periods. Most studies reporting a competitive advantage of *P. aeruginosa* use prolonged culture times, emphasizing the dynamic nature of these interactions [20, 78, 84, 88, 113]. These findings are subject to limitations inherent to experimental conditions and strain‐specific interactions. Culture conditions (e.g., static vs. mixed cultures) influence the competitive or cooperative dynamics observed. For example, Barraza et al. report a reduced antagonistic effect under anoxic conditions provided by static culture [78]. However, in a study performed by Sánchez‐Peña et al., similar conditions were employed, and an antagonistic effect was seen [88].

These data suggest that adaptation to the CF microenvironment allows both pathogens to transition to cooperative behaviors and coexist. It is still puzzling, however, why *P. aeruginosa* and *S. aureus* are more often coisolated in the initial stages of CF disease, while *P. aeruginosa* predominates in later adulthood. Further investigations beyond the present understanding of temporal dynamics and their clinical implications are required.

### 2.2. Interactions of *P. aeruginosa* With *Candida* spp.

Among fungal members of the CF lung microbiome, *Candida* spp. have received significant attention in CF biofilm studies. A Swedish cohort of 186 CF patients found *Candida* spp. to be more frequently coisolated with *P. aeruginosa* in CF patients compared to those with other respiratory diseases, though its overall frequency remained low [114]. This suggests possible strain‐specific cooperation or antagonism between these organisms.

A study by Alam et al. showed an increase in resistance to meropenem in biofilm cocultures of *P. aeruginosa* and *Candida* spp. This increased resistance was attributed to interactions between *Candida albicans* mannans and glucans with *P. aeruginosa* [91]. A separate study exposed culture extracts of *C. albicans* and *Candida parapsilosis* to *P. aeruginosa* and found decreased susceptibility to meropenem but increased susceptibility to cephalosporins, colistin, and tobramycin [92]. Transcriptomic analyses by the group of Alam et al. further revealed upregulation of *P. aeruginosa* efflux pump and biofilm‐related genes during coculture with *C. albicans*, possibly contributing to reduced antibiotic efficacy [21].

On the other hand, *P. aeruginosa* can antagonize *Candida* spp. through PYO‐mediated damage to fungal cell walls, particularly in the hyphal form, and inhibition of the yeast‐to‐hyphae transition by 3‐oxo‐C12‐HSL [115]. In turn, *Candida* produces farnesol, which inhibits the expression of *P. aeruginosa* virulence factors [22, 115]. This interaction is strain‐specific, since *P. aeruginosa lasR* mutants exposed to farnesol had increased phenazine biosynthesis compared to wild‐type strains in this study [22]. Additionally, iron competition mediated by *P. aeruginosa* siderophores reduces iron availability and limits fungal growth [115].

### 2.3. Interactions of *P. aeruginosa* With *Aspergillus* spp.

*Aspergillus fumigatus* is the most isolated filamentous fungi species across all age groups of pwCF, with occasional cocolonization with *P. aeruginosa* [77, 116–118]. Cocolonization rates and associated outcomes vary across studies. A 749‐patient cohort study reported a low cocolonization rate (3.81%), which was also associated with poorer outcomes [119]. In contrast, a meta‐analysis of 2114 patients estimated a prevalence of 15.8% [116], while a larger UK cohort of 9270 patients reported a cocolonization rate of 9.1%, with no observed difference in disease severity compared to patients colonized solely with *P. aeruginosa* [118].

Biofilm coculture studies show that *P. aeruginosa* and *A. fumigatus* engage in antagonistic interactions. *P. aeruginosa* increases elastase [93], rhamnolipids, pyoverdine, and biofilm synthesis in the presence of *A. fumigatus*, a response driven by phenazines [94]. One study reported that phenazines also inhibit fungal germination by disrupting mitochondrial redox balance and inducing ROS production. Specifically, 1‐hydroxyphenazine (1‐HP) enhances the toxicity of other phenazines by chelating iron. In this same study, gliotoxin produced by *A. fumigatus* was proposed as a defensive mechanism against *P. aeruginosa,* supported by the increased phenazine sensibility observed in a gliotoxin‐deficient *A. fumigatus* mutant [94]. Interestingly, the production of PYO, phenazine‐1‐carboxylic acid, and phenazine‐1‐carboxamide facilitates iron acquisition by *A. fumigatus* through iron reduction [95].

Consistent with these findings, a study by Sass et al. demonstrated that iron depletion mediated by pyoverdine inhibited *A. fumigatus*, particularly in its planktonic state [96]. However, siderophore‐deficient *P. aeruginosa* mutants also show antifungal activity due to increased production of rhamnolipids and elastase under iron‐depleted conditions [97]. Since the antagonism of *P. aeruginosa* toward *A. fumigatus* involves increased secretion of virulence factors, exposure to *A. fumigatus* in vivo might increase cytotoxicity toward the host [93].

All the aforementioned interactions underscore the antagonistic effects of *P. aeruginosa* on *A. fumigatus*. This behavior seems to be kept independent of oxygen and iron availability [94, 96]. A notable exception to the antagonistic behavior toward *A. fumigatus* was identified by Briard et al., who reported a novel airborne interaction involving dimethyl sulfide produced by *P. aeruginosa* that stimulates *A. fumigatus* growth, revealing an alternative pathway for microbial communication [98].

### 2.4. Interactions With Commensal Bacteria

Recent evidence underscores a crucial role of commensal bacteria in the progression of lung disease in pwCF. A higher relative abundance of commensals is associated with milder lung disease [120, 121], likely due to their nonspecific anti‐inflammatory effects [99, 100]. In contrast, chronic *P. aeruginosa* colonization is associated with reduced microbial diversity and relative abundance of commensals, a trend linked to poorer clinical outcomes [106]. Although the protective effects of commensals are often nonspecific, increasing research highlights a complex interplay and cross‐communication between commensal bacteria and *P. aeruginosa*.

*Prevotella* spp., commonly isolated from CF airways, has shown a range of interactions with *P. aeruginosa*. Bertelsen et al. demonstrated that *Prevotella* species exerted anti‐inflammatory effects in a mixed infection model using CF bronchial epithelial cells. This anti‐inflammatory effect was mediated by the inhibition of *P. aeruginosa* growth through an unidentified, secreted, heat‐stable compound [101]. Similarly, Grassi et al. found that several *Prevotella* species exhibited inhibitory and antibiofilm activities against *P. aeruginosa*; this study also reported suppression of virulence factor production in *P. aeruginosa* [102]. Not all interactions are antagonistic, as *Prevotella* species harboring beta‐lactamases can indirectly protect *P. aeruginosa* from beta‐lactams [103]. These findings underscore the dual roles that *Prevotella* spp. may play in CF airways, ranging from protective to potentially harmful, depending on environmental and microbial contexts.

Similarly, *Rothia mucilaginosa*, another commonly isolated anaerobe from CF airways, has shown anti‐inflammatory effects when cocultured with *P. aeruginosa*. Rigatus et al. reported that *R. mucilaginosa* exerts a nonspecific anti‐inflammatory effect, reducing the secretion of proinflammatory cytokines triggered by the immune recognition of *P. aeruginosa*. This effect was observed under microaerobic conditions, which mimic the CF lung microenvironment. Additionally, the abundance of *R. mucilaginosa* in sputum samples was negatively correlated with proinflammatory cytokine levels in the sputum [99].

In terms of microbial dynamics, *Rothia* species exhibit mild inhibitory effects on *P. aeruginosa* growth, as noted by Tony‐Odigie et al., although the mechanisms behind this interaction remain unclear [104]. Furthermore, Gao et al. described a metabolic interaction in which *P. aeruginosa* utilizes lactate produced by *R. mucilaginosa* as a carbon source for amino acid biosynthesis [105]. Collectively, these findings suggest that *R. mucilaginosa* plays a multifaceted role in the CF lung environment, not only modulating the host immune response but also engaging in metabolic interactions with *P. aeruginosa*, which may influence microbial dynamics and disease progression.

Despite these intricate interspecies interactions, transcriptomic studies show that gene expression of *P. aeruginosa* is minimally affected when cocultured with commensal bacteria. For example, in a coculture study involving *S. aureus*, *S. sanguinis*, and *Prevotella melaninogenica*, the transcriptomic profile of *P. aeruginosa* had minor changes [122]. Similarly, a study by Barraza et al. observed limited transcriptomic alteration in *P. aeruginosa* during coculture with *S. aureus* alone [90]. These suggest that while gene expression of other species is highly influenced by the CF lung dynamics and immune responses, *P. aeruginosa* remains relatively transcriptionally resilient in the presence of these species.

## 3. Methods to Study *P. aeruginosa* Biofilms in CF Research

Methods for studying bacterial biofilms are routinely applied to CF research, while others were specifically developed to mimic *P. aeruginosa* in CF‐like conditions [123]. In this section, we provide a brief overview of the most commonly used methods and techniques to study biofilms in CF research [123].

### 3.1. Direct Visualization of Biofilms From Clinical Samples

Biofilms can be visualized in clinical samples using routine stains such as Gram, hematoxylin–eosin, and alcian blue. Although these methods lack specificity for *P. aeruginosa*, they combined provide a general overview of biofilm architecture and its interactions with host cells and tissues [25, 124, 125]. Such approaches have also been used to validate specialized experimental models, including in vivo mouse models [126] and the ex vivo pig lung model [127].

Specific detection of *P. aeruginosa* within biofilms can be achieved using fluorescent in situ hybridization (FISH) with rRNA‐targeted probes [25]. Matrix components are commonly visualized with fluorescently labeled antibodies [28, 29]. A more advanced technique, the Microbial Identification after Passive CLARITY Technique (MiPACT), enables preservation of ex vivo biofilm structures by embedding clinical specimens in a hydrogel matrix. This is followed by tissue clarification and subsequent immunofluorescence and/or FISH [123].

### 3.2. In Vitro Systems for Biofilm Formation

#### 3.2.1. Microplate Culture

Biofilm development in multiwell microplates is widely used in CF research due to its simplicity, versatility, and high‐throughput, and it often serves as the initial step for many downstream applications [128–133]. In this approach, microplates containing liquid media are incubated to allow biofilms to develop on the well surfaces. After incubation, planktonic cells in the liquid fraction are removed, and the attached biofilm is retained for subsequent analyses. Variations in well size and plate format generally have minimal impact on assay outcomes, provided that the bacteria‐to‐broth ratio remains constant [20, 90, 91, 129–132, 134]. A common variation of this method employs peg‐lid systems, known as the Calgary biofilm device, in which biofilms form on the pegs and are transferred to fresh microplates for further analyses [135].

#### 3.2.2. Microfluidic Systems

Microscopic flow cell systems provide an effective means to overcome the inherent limitations of static microplate cultures. These platforms maintain a continuous flow of fresh medium, enabling the study of biofilm development under controlled conditions while requiring minimal sample volumes. A major advantage of this approach is its compatibility with confocal laser scanning microscopy (CLSM), which allows dynamic visualization of biofilms. Additionally, flow cells facilitate the introduction of external agents such as antibiotics or potential molecules with antibacterial potential, allowing the assessment of biofilm responses to external factors [27, 69, 70, 89, 136–138]. However, biofilms formed under continuous flow may differ from the static, oxygen‐limited, and nutrient‐restricted biofilms in chronically infected CF lungs.

#### 3.2.3. Optimization of Culture Media: Synthetic Cystic Fibrosis Sputum Media (SCFM)

The choice of culture medium used in in vitro studies impacts experimental outcomes, as medium composition influences the transcriptomic profile of *P. aeruginosa* [139]. Considerable efforts have been dedicated to developing synthetic media that accurately replicates the nutritional and physicochemical properties of CF mucus, leading to the development of artificial sputum media (ASM). Neve et al. demonstrated that the composition of different ASM formulations influences *P. aeruginosa* metabolite production, primarily due to substrate availability. Among the tested formulations, the Synthetic Cystic Fibrosis Sputum Medium 2 (SCFM2) and the variant SCFM3, modified versions of ASM, induced the highest production of phenazines, particularly PYO, whereas other formulations yielded higher proportions of 1‐HP [139]. Media composition also affected quinolone production: SCFM2 and SCFM3 supported the production of HQNO, HHQ, and PQS in proportions similar to those found in CF sputum [140, 141]. Additionally, rhamnolipid levels in these formulations were consistent with in vivo measurements [141], whereas pyoverdine synthesis occurred exclusively in SCFM1. As the study of Neve et al. used only the reference strain PAO1, these results should be interpreted cautiously when extrapolating to clinical or environmental isolates. Another advantage of using ASM formulations is their ability to reproduce the enhanced antibiotic susceptibility observed in vivo in CF sputum [142–145], further underscoring their value as physiologically relevant models for CF biofilm research.

SCFM2 has proven to be the most effective in reproducing in vivo‐like conditions. Comparative transcriptomic analyses of *P. aeruginosa* grown in SCFM2, CFBE cells, mouse lungs, MOPS‐succinate, and LB broth, relative to in vivo sputum samples, showed that SCFM2 most closely replicated in vivo gene expression profiles, especially for CF‐adapted isolates, achieving up to 90% similarity with the in vivo transcriptome [146]. Furthermore, SCFM2 has been shown to efficiently reproduce the metabolic activity observed in vivo sputum, encompassing pathways identified as essential under CF conditions through transposon mutant analyses [147].

### 3.3. Study of Structural Aspects of *P. aeruginosa* Biofilms

Raw biofilm formation can be evaluated using the microplate and Calgary device methods by staining the attached growth using crystal violet [128]. Although these approaches are scalable, they primarily quantify total biomass and provide limited information about the internal structure and composition of biofilms. Conventional immunofluorescence microscopy and CLSM remain the most widely used techniques for in vitro investigation of biofilm architecture and components. As with direct visualization methods, fluorescently labeled antibodies are employed to detect specific matrix components [29], while the spatial distribution of bacteria within biofilms can be examined using genetically engineered strains expressing fluorophores [69, 88]. The integration of CLSM with microfluidic chambers allows longitudinal analyses of cellular organization within biofilms, while allowing controlled administration of antimicrobial agents to assess biofilm responses [27, 67, 68, 93].

### 3.4. Viability Assessment From Biofilms

Determining the viability of cells within biofilms is essential for evaluating the effects of antibiotic exposure and the efficacy of antimicrobial agents. The minimum biofilm eradication concentration (MBEC), which is the antibiotic concentration necessary to eradicate an established biofilm, and the biofilm prevention concentration (BPC), which evaluates the concentration of antibiotic needed to avoid biofilm formation, were developed to account for the increased tolerance associated with biofilm growth [14]. The Calgary biofilm device enables high‐throughput evaluation of both parameters by transferring peg‐grown biofilms to microplates containing fresh antibiotic solutions and subsequently measuring optical density at 600 nm following antibiotic exposure [135, 137]. Viability can also be assessed through serial dilution and CFU enumeration after treatment; however, these methods are less robust due to potential errors during the planktonic cell removal step and are not easily scalable [14, 91, 136]. Nonetheless, the CFU count is suitable for evaluating interspecies competition by performing cocultures and subsequently plating in species‐specific media [20, 84].

Beyond CFU recovery and enumeration, cell viability within biofilms can be assessed using differential fluorescent staining methods that distinguish live from dead cells, such as the BacLight LIVE/DEAD assay [148] and flow cytometry [64]. The ratio of viable or nonviable cells can be quantified based on the fluorescence intensity of each dye or directly visualized through microscopy [138, 149]. Alternatively, the redox indicator resazurin can be used to detect metabolically active cells through reduction to resorufin, which is associated with a characteristic pink color change [142]. Furthermore, a recent rRNA‐based RT‐qPCR approach has been proposed, enabling the quantification of viable cells within mixed cultures using species‐specific primers [150]. Altogether, these viability‐based methods offer a significant advantage over culture‐dependent techniques, as they allow the detection of metabolically active subpopulations.

### 3.5. Specialized Models

Most methods employed in CF research have roots outside of this context; however, there are specialized models developed to mimic CF‐like conditions. These have allowed the study of complex properties, such as host–microbe and polymicrobial interactions. Below we list some of the most relevant models:

a. Epithelial cell line models, such as CF‐derived bronchial epithelial cells (CFBEs) [80, 151–153] or cell lines carrying the F508del mutation [83], effectively simulate in vivo airway conditions. These models allow CFU quantification, live imaging of microbial interactions, and cytotoxicity assessments against host cells [152, 153]. This method offers essential insights into microorganism–host interactions and therapeutic responses.
b. In vivo models, mostly mouse models, are used to replicate CF lung environments [154]. Approaches include CFTR knockout strains [155, 156], overexpression of the *β*‐subunit of the epithelial sodium channel [157], and immune suppression [158]. While useful for studying bacteria–immune system interactions, these models often yield variable results and only partially recapitulate key features of CF pathophysiology [158]. Less commonly used animal models, such as pigs, ferrets, rats, and zebrafish, provide alternative systems but have limited applicability for *P. aeruginosa* studies [154].
c. Vandeplassche et al. developed a method to investigate contact‐independent biofilm interactions, wherein *P. aeruginosa* biofilms are grown on polyvinylidene fluoride filters placed above receiver plates containing other CF‐associated microorganisms. This design allows for media exchange without direct physical contact, enabling the study of microbial interactions while avoiding physical interference [159].
d. An ex vivo pig lung model developed by Harrington et al. [127] was recently proposed. This model overcomes limitations of traditional in vivo systems and is considered both ethical and a high‐throughput model. It successfully replicates hallmark biofilm characteristics, including enhanced antibiotic tolerance [160] and in vivo‐like transcriptomic profiles [161].

## 4. Antibiofilm Therapeutic Strategies

The transition from a planktonic to a biofilm lifestyle is a key factor contributing to the persistence of *P. aeruginosa* in pwCF, as it enhances resistance to antibiotics. Antibiofilm strategies are aimed at disrupting this resilient lifestyle, targeting biofilm formation, maintenance, and dispersal to mitigate chronic infections effectively [43, 47].

### 4.1. Combination of Antibiotics

The use of antibiotic combinations has been investigated as a potential antibiofilm treatment. Sequential administration of tobramycin and aztreonam has demonstrated enhanced biofilm clearance compared to monotherapy, likely due to synergistic effects on biofilm penetration and bacterial eradication [162]. Emerging studies are also focusing on optimizing dosing regimens and exploring novel combinations to improve antibiotic efficacy against biofilms. For example, a study combining antibiotics with organic acids, such as ciprofloxacin with D, L‐malic acid and ceftazidime with sodium acetate, significantly inhibited biofilm growth and eradicated established biofilms. These effects were mediated by modulation of bacterial metabolism, including increased TCA cycle activity, pH alterations, and ROS production. However, the observed effects were strain‐specific, underscoring the importance of tailored therapeutic approaches [163].

### 4.2. Disruption of Biofilm

Biofilm eradication necessarily involves disruption of the EPS matrix, as it forms a physical barrier that protects bacterial cells against antibiotics and immune responses. Agents targeting the EPS matrix can be classified into specific and nonspecific matrix disruptors. The focus of specific therapies has been on the degradation of specific EPS components. For example, *P. aeruginosa* endogenously produces glycoside hydrolases, such as Psl and Pel (PslG_h_ and PelA_h_), which target and degrade matrix EPS. Alginate lyases themselves are well studied and can be sourced from a variety of organisms, including algae [47, 164]. Addition of such enzymes enhances the penetration and efficacy of antibiotics like tobramycin and ciprofloxacin [165]. Although not inherently bactericidal, these agents have an adjuvant role in supporting traditional antibiotics, underscoring the importance of combination therapies that could effectively achieve biofilm eradication.

Andersen et al. showed that the activation of endogenous PDEs promotes biofilm dispersal by reducing c‐di‐GMP levels [166]. This finding has facilitated the development of novel synthetic molecules, such as H6‐335 and H6‐335‐P1, which specifically target PDEs. Both in vitro experiments and in vivo studies using mouse models have shown that activating endogenous PDEs promotes biofilm dispersal. Notably, when combined with commonly used antibiotics, such as tobramycin and ciprofloxacin, this approach led to increased efficacy [167].

Another strategy involves using QS‐molecule analogs, such as L‐HSL (N‐[3‐cyclic butyrolactone]‐4‐trifluorophenylacetamide), which inhibit biofilm formation and disrupt preformed biofilms by downregulation of QS‐dependent pathways [168]. Finally, AMPs have been evaluated in combating multidrug‐resistant *P. aeruginosa* biofilms isolated from pwCF. One study using synthetic peptides (D, L‐K6L9) reported robust antimicrobial and antibiofilm activity and reduced biofilm biomass in clinical and artificial sputum conditions. Unlike conventional antibiotics, the D, L‐K6L9 peptides were resistant to degradation by CF sputum proteases and did not promote the development of bacterial resistance [169].

### 4.3. Bacteriophages

Bacteriophage therapy is a promising approach for biofilm dispersal. Phages can disrupt biofilms by degrading the ECM, thereby facilitating antibiotic penetration. The use of phage cocktails enhances antibiofilm efficacy by preventing resistance development and broadening host specificity. Despite these advantages, challenges such as the emergence of phage resistance and biofilm density emphasize the need for further optimization and integration with complementary therapeutic strategies [170, 171].

### 4.4. Nanomedicine

The conjugation of existing antibiotics with nanostructures has shown potential for improving antibiotic uptake within biofilm structures. Liquid‐crystal nanoparticles conjugated with tobramycin and hydrophobic molecules, such as monoolein, enhance biofilm targeting while minimizing interaction with epithelial cells [172]. Additionally, silver nanoparticles (AgNPs) have intrinsic antibiofilm and biofilm‐disrupting activity, induce oxidative stress, and disrupt iron homeostasis [173]. Transcriptomic studies have further demonstrated that AgNPs downregulate the expression of QS system genes [174]. Similarly, nanocarriers encapsulating antibiotics and coated with matrix‐disrupting enzymes, such as alginate lyases, elastases, and DNAses, have shown promising results in in vivo mouse models [175].

## 5. Concluding Remarks

The persistence of *P. aeruginosa* biofilms in CF airways is a significant challenge in clinical management due to their robust structure, adaptability, and interactions within the polymicrobial environment. This review highlights the intricate mechanisms that sustain *P. aeruginosa* biofilms, including the pivotal roles of exopolysaccharides, QS systems, and pathoadaptive changes unique to the CF lung microenvironment. Additionally, the complex synergistic and antagonistic interactions with other microbes, such as *S. aureus*, underscore the complexity of these infections and the necessity for innovative therapeutic strategies. Although emerging antibiofilm approaches such as matrix‐targeting enzymes, QS inhibitors, and novel drug delivery systems show significant promise, their efficacy must be validated in models that closely mimic CF‐like conditions. Moreover, a deeper understanding of microbial interactions within the diverse CF microbiome may uncover novel avenues to weaken biofilm resilience and improve patient outcomes.

## Funding

No funding was received for this manuscript.

## Conflicts of Interest

The authors declare no conflicts of interest.

## Data Availability

Data sharing is not applicable to this article as no datasets were generated or analyzed during the current study.
